# WHO Framework Convention on Tobacco Control Learnings

**DOI:** 10.1089/hs.2023.0094

**Published:** 2023-09-26

**Authors:** Joanna E. Cohen, Matthew L. Myers, Indu B. Ahluwalia

**Affiliations:** Joanna E. Cohen, PhD, is Director, Institute for Global Tobacco Control, and Bloomberg Professor of Disease Prevention, Department of Health, Behavior and Society, Johns Hopkins Bloomberg School of Public Health, Baltimore, MD.; Matthew L. Myers, JD, is Former President, Campaign for Tobacco-Free Kids, Washington, DC.; Indu B. Ahluwalia, PhD, is Branch Chief, Global Tobacco Control Branch, Office on Smoking and Health, National Center for Chronic Disease Prevention and Health Promotion, Centers for Disease Control and Prevention, Atlanta, GA. The findings and conclusions in this document are those of the authors and do not necessarily represent the official position of the Centers for Disease Control and Prevention.

**Keywords:** Epidemic management/response, Regulatory issues < legal aspects, Policy, Countermeasures, Citizen engagement

We are pleased that the World Health Organization (WHO) is developing a pandemic treaty to improve the global response to future pandemics. In their article, De Luca and Ramirez^1^ rightly argue that a pandemic treaty should be informed by experiences with WHO's existing treaty, the WHO Framework Convention on Tobacco Control (WHO FCTC), which came into force in 2005. However, the authors have mischaracterized the WHO FCTC and made criticisms that could hinder a productive discussion about a treaty for pandemics.

The authors identify a limited emphasis on “harm reduction” as a key limitation of the WHO FCTC. However, the treaty itself includes “harm reduction strategies” in its definition of tobacco control.^2^ As the authors indicate, harm reduction encompasses actions “aimed at reducing the negative effects of health behaviors without necessarily extinguishing the problematic health behaviors completely or permanently.”^1,3^ The WHO FCTC and its guidelines for implementation—which include requiring smoke-free public places; banning tobacco advertising, promotion, and sponsorship; and reducing the attractiveness of tobacco products by limiting flavoring agents—allow the continuation of product use, while reducing the negative effects. Further, the treaty applies to all tobacco products, including products beyond cigarettes. The Conference of the Parties to the WHO FCTC, the governing body of the Convention, has outlined measures that Parties should prioritize to address the challenge of tobacco products, such as heated tobacco products,^4^ as well as policy objectives and options for Parties to consider in regulating electronic nicotine delivery systems and electronic non-nicotine delivery systems.^5-8^

It is also crucial to point out that the relationship between the tobacco industry and tobacco-caused death and disease is very different than the relationship of pharmaceutical and other companies to an infectious disease pandemic. The tobacco industry's own products are responsible for the deaths and diseases the treaty addresses. This is not the case with a pandemic caused by an infectious agent. Tobacco companies are driven to sell their products and find new users, which is a fundamental and irreconcilable conflict with the interests of public health policy.^9^ And, tobacco companies continue to introduce products that they claim will reduce harm to sell tobacco products and expand their user base by distorting and concealing the evidence on the harmful effects of these and other tobacco products.^8,10-12^

Tremendous progress has been made in addressing the tobacco pandemic. The implementation of the WHO FCTC has helped to save more than 37 million lives and counting,^8^ and the global prevalence of tobacco use has declined from almost 33% in 2000 to 22% in 2020.^13^ Certainly, greater progress can be made in reducing tobacco use across the globe, however, a substantial impediment remains: tobacco companies aggressively fight whenever countries attempt to implement the WHO FCTC.^12,14-16^

It will be essential for a pandemic treaty to address anticipated challenges from its inception. Harm reduction and private-sector involvement, and its impact in low- and middle-income countries, may very well be among those challenges; however, these issues present very differently from the WHO FCTC. The WHO FCTC has demonstrated the powerful impact a treaty can have on public health when it articulates clear, evidenced-based measures whose benefits have been proven and documented in different political systems and cultures across the globe and whose public health outcomes can be measured objectively. Critical to the WHO FCTC's success has been an organized, sustained, coordinated, proactive civil society that has been involved from the beginning of treaty negotiations through implementation, which has helped overcome inertia and counter industry opposition that is more interested in sustaining its profits than protecting public health.
